# Nebulized Tranexamic Acid in Secondary Post-Tonsillectomy Hemorrhage: Case Series and Review of the Literature

**DOI:** 10.5811/cpcem.2021.5.52549

**Published:** 2021-07-27

**Authors:** Mira Dermendjieva, Anand Gopalsami, Nicole Glennon, Sam Torbati

**Affiliations:** * Cedars Sinai Medical Center, Department of Pharmacy Los Angeles, California; † Cedars Sinai Medical Center, Department of Emergency Medicine, Los Angeles, California

**Keywords:** tonsillectomy, hemorrhage, nebulized tranexamic acid

## Abstract

**Introduction:**

Post-tonsillectomy hemorrhage is a serious postoperative complication, and its acute management can present a challenge for the emergency provider. Although various strategies have been proposed, guidance on the best approach for management of this condition in the emergency department (ED) setting remains limited. Anecdotal reports of the use of nebulized tranexamic acid (TXA) for management of tonsillar bleeding have emerged over the past two years. Two recently published case reports describe the successful use of nebulized TXA for stabilization of post-tonsillectomy hemorrhage in an adult and a pediatric patient.

**Case Series:**

Eight patients who presented to our ED with secondary post-tonsillectomy hemorrhage received nebulized TXA for hemostatic management. The most common TXA dose used was 500 milligrams, and all but one patient received a single dose of the medication in the ED. Hemostatic benefit was observed in six patients, with complete bleeding cessation observed in five cases. Interventions prior to nebulized TXA administration were attempted in three of the six patients and included ice water gargle, direct pressure with TXA-soaked gauze, and nebulized racemic epinephrine. All but one of the patients were taken to the operating room for definitive management after initial stabilization in the ED.

**Conclusion:**

Nebulized TXA may offer a hemostatic benefit and aid in stabilization of tonsillectomy hemorrhage in the acute care setting, prior to definitive surgical intervention. Consideration of general principles of nebulization and aerosol particle size may be an important factor for drug delivery to the target tissue site.

## INTRODUCTION

Post-tonsillectomy hemorrhage is the most common serious complication of tonsillectomy, generally thought to occur at a rate of 0.1–3%.^1^,^2^ The majority of patients who encounter this complication experience secondary post-tonsillectomy hemorrhage, which is defined as bleeding beyond 24 hours post-surgery. It typically occurs between postoperative days four and 10, as tonsillar eschar sloughs off.^1^ Guidance is limited on the best approach for management of post-tonsillectomy bleeding in the emergency department (ED) setting. Strategies deployed for management of this condition in the ED include cold fluids, direct pressure, clot suction, silver nitrate, topical epinephrine, thrombin powder and – more recently – nebulized tranexamic acid (TXA).^3^,^4^,^5^

Published literature on the use of inhaled TXA for this indication is currently limited to two case reports: one pediatric and one adult.^4^,^5^ Despite the absence of controlled trials, extrapolation from correlate data has been deemed reasonable by some providers.^6^–^12^ Based on existing data of the use of inhaled TXA in management of hemoptysis, the drug appears to have an excellent safety profile when given via nebulization. It is also easy to use, relatively quick to administer, low in cost, and requires no airway manipulation for delivery. This case series aims to add to existing case report literature on the use of nebulized TXA for management of post-tonsillectomy bleeding in the ED, as well as to review the basis of TXA use for this indication.

## CASE SERIES

### Case 1

A 43-year-old female with past medical history significant for obstructive sleep apnea, Ehlers-Danlos syndrome and obesity presented to the ED for oropharyngeal bleeding. She was six days status post tonsillectomy, uvulopalatopharyngoplasty, revision septoplasty, and bilateral inferior turbinate resection. Examination of her airway revealed postoperative granulation tissue, bright red blood, and a large clot in her posterior oropharynx, which was occluding most of her airway. Control of bleeding was first attempted with direct pressure using packing gauze soaked in TXA 500 milligrams (mg) per five milliliters (mL) solution. This was followed by nebulized racemic epinephrine 2.25% 0.25 mL, immediately followed by nebulized TXA 1000 mg / 10 mL. No active bleeding was noted upon arrival of the patient’s ear, nose and throat (ENT) surgeon within 30 minutes of the interventions. She was taken to the operating room (OR) where a blood clot from the right inferior tonsillar pole was evacuated with subsequent successful cauterization of the bleeding site.

### Case 2

A 13-year-old female with past medical history significant for obstructive sleep apnea secondary to adenotonsillar hypertrophy, recurrent epistaxis, and menometrorrhagia presented to the ED for hematemesis. She was seven days status post bilateral adenotonsillectomy. On examination of her airway, minimal oozing from the posterior oropharynx was noted. Tranexamic acid 500 mg / 5 mL diluted with 5 mL of normal saline was administered via nebulizer. On arrival of the ENT surgeon, about 40 minutes post-TXA administration, the patient was still noted to have bleeding from the mouth and a clot on the right superior tonsillar pole. She was taken to the OR where a large blood clot, partially obstructing her larynx, was suctioned and a bleeding point in the right lower tonsil was successfully cauterized. The patient completed an outpatient hematology workup, which did not identify a hematologic abnormality.

### Case 3

A 29-year-old male with past medical history significant for chronic tonsillitis presented to the ED following an episode of hemoptysis, four days status post bilateral tonsillectomy. Initial examination of the airway revealed displacement of the left tonsillar eschar by a large blood clot. The patient continued to experience small volume hemoptysis in the ED and was taken to the OR where hemostasis was attained via cauterization. He returned to the ED five days later, on postoperative day nine, for recurrent tonsillar bleeding. On examination of the airway, a left tonsillar clot was again visualized. Nebulized TXA 500 mg / 5 mL was administered, and the patient was admitted to the hospital for observation. No further bleeding was reported. Nebulized TXA 500 mg / 5 mL was continued for two additional doses, administered 12 hours apart during his short inpatient stay. Given the recurrent bleeding episodes, hematologic studies were subsequently performed with no overt hematologic abnormality identified.

**CPC-EM Capsule**

What do we already know about this clinical entity?

*Post-tonsillectomy hemorrhage is a serious postoperative complication, and guidance on management in the emergency department (ED) remains limited*.

What makes this presentation of disease reportable?

*This case series describes the use of nebulized tranexamic acid (TXA) in the ED to achieve hemostasis in post-tonsillectomy hemorrhage*.

What is the major learning point?

*Nebulized TXA may offer a hemostatic benefit and aid in stabilization of tonsillectomy hemorrhage in the ED setting prior to definitive surgical intervention*.

How might this improve emergency medicine practice?

*Given the limited therapeutic options, nebulized TXA may be a safe, non-invasive option for short-term stabilization pending definitive surgical intervention*.

### Case 4

A 17-year-old male with past medical history significant for recurrent sinus infection, tonsillar hypertrophy, and chronic tonsillitis presented to the ED with complaint of oropharyngeal bleeding. He was five days status post bilateral adenotonsillectomy and nasal septoplasty. Examination of the airway confirmed the presence of active bleeding from his posterior oropharynx. Nebulized TXA 500 mg / 5 mL was administered, followed by an ice water gargle. Each of the two interventions appeared to aid in decreasing the amount of bleeding; however, significant bleeding from his posterior oropharynx soon resumed. The patient was taken to the OR where an area of arterial bleeding was identified and successfully cauterized.

### Case 5

A 23-year-old male with no pertinent past medical history presented to the ED with a complaint of oropharyngeal bleeding, which had been unrelieved by application of ice chips at home. He was 13 days status post bilateral tonsillectomy and had undergone outpatient cauterization of the left tonsillar base for recurrent bleeding earlier that day. Examination of the airway revealed a clot and active bleeding at the left palatine tonsillar fossa. Ice water gargle was first attempted, followed by suctioning of some of the clot and a dose of nebulized TXA 500 mg / 5 mL. These interventions did not completely abate the bleeding and were followed by an additional dose of TXA 500 mg / 5 mL sprayed directly onto the bleeding site with a mucosal atomizer device. Hemostasis was only temporarily achieved. Next, oxymetazoline and lidocaine 4% solution were applied topically to anesthetize the area and allow local injection of lidocaine 1% plus epinephrine. Direct pressure was then applied for five minutes. Hemostasis was briefly attained, but oozing soon resumed. The patient was taken to the OR for definitive control of bleeding via cauterization of the tonsillar beds.

### Case 6

A six-year-old male with past medical history significant for tonsillar hypertrophy was brought to the ED for oropharyngeal bleeding and an episode of hematemesis 20 minutes prior to arrival. He was six days status post bilateral tonsillectomy and adenoidectomy. On examination of his airway, active posterior oropharyngeal bleeding was noted without a specific source identified. The patient was instructed to perform an ice water gargle, which was followed by a dose of nebulized TXA 500 mg / 5 mL. Some oozing was still notable immediately following the nebulization; however, on arrival of the ENT surgeon approximately 30 minutes later no active bleeding was observed. The patient was taken to the operating room where a large clot on the right tonsillar pole was suctioned, revealing a few punctate areas of oozing that were successfully cauterized.

### Case 7

A 13-year-old female with past medical history significant for tonsillar adenoid hypertrophy and chronic tonsillitis presented to the ED with complaints of hemoptysis with clots. She was 12 days status post bilateral tonsillectomy and adenoidectomy. On examination of her airway, active bleeding from the posterior pharynx was observed. Nebulized TXA 500 mg / 5 mL diluted in 5 mL normal saline was administered. The patient reported feeling better following the nebulization, and repeat examination of the airway revealed hemostasis had been achieved. She was subsequently taken to the OR where a clot over the right tonsillar pole was observed along with recurrence of bleeding in the same region. The area was cauterized and microfiber collagen hemostat powder applied over the right tonsillar fossa.

### Case 8

A 24-year-old male with past medical history significant for chronic tonsillitis presented to the ED with complaint of recurrent tonsillar bleeding 15 days status post bilateral tonsillectomy. This was the third episode of bleeding he had experienced postoperatively, with the first occurrence necessitating a return to the OR for left tonsillar cauterization on postoperative day nine. The second episode had been successfully managed in the ED with bedside silver nitrate cauterization of the right tonsil on postoperative day 14. Upon presentation of this third recurrence, examination of the patient’s airway revealed bilateral tonsillar bleeding. Nebulized TXA 1000 mg / 10 mL was administered. Cessation of bleeding and clot formation were noted on re-examination within 20 minutes of completion of the nebulization. The patient was taken to the OR where a right tonsillar clot was suctioned and the right middle tonsillar pole successfully cauterized. Six days later, the patient reported a brief episode of a small amount of recurrent bleeding, which self-resolved. No further bleeding was reported thereafter.

A summary of the patients’ characteristics, interventions and outcomes is shown in the table below. Coagulation parameters and platelet counts were assessed in all patients and were found to be within normal limits. There was no history of blood dyscrasias or overt suspicion for bleeding disorder in any patient, although two patients subsequently underwent negative outpatient hematology workups. There was no reported use of anticoagulants or antiplatelet agents in any patient. No hemodynamic instability, airway compromise or need for blood transfusion related the tonsillar hemorrhage occurred in any patient within this cohort.

## DISCUSSION

Since its discovery and introduction in the 1960s by Japanese scientists Utako and Shosuke Okamoto, TXA has been used to aid the management of bleeding in a wide range of medical, dental, and surgical settings and among diverse patient populations.^13^–^15^ Pharmacologically TXA is a synthetic antifibrinolytic agent that prevents the breakdown of the polymerized fibrin clot matrix. Fibrinolysis is part of the usual, complex processes that comprise vascular hemostasis. It involves activation of circulating plasminogen by tissue plasminogen activator into plasmin, which subsequently degrades existing fibrin clot.^16^ For its role, TXA competitively inhibits the lysine-binding site on plasminogen, preventing it and subsequently formed plasmin from interacting with lysine residues on the fibrin polymer, thereby subverting fibrinolysis and subsequent clot degradation.^17^ The degree of inhibition of fibrinolysis by TXA has been described as being concentration dependent.^18^,^19^

While TXA has traditionally been used via the oral, intaravenous (IV) and topical route, the past decade has seen the exploration of aerosolized application of TXA to anatomically sequestered areas such as the lungs and posterior nasal cavity.^10^–^26^ It has been hypothesized that TXA may be particularly effective for management of oropharyngeal bleeding because of the relatively high concentration of plasminogen and low concentration of intrinsic plasminogen inhibitors found in saliva.^4^,^22^ Aerosolization of the drug may also provide key advantages including the ability to reach anatomically sequestered sites and perhaps allow for better delivery of sufficient drug concentrations to the active site of bleeding when compared to other routes of administration.

Although pharmacokinetic studies of nebulized TXA are not yet available, correlate data from other experiments provides some useful insights. Topical application of 5% TXA solution via two-minute 10 mL mouth rinse was found to achieve average salival concentration three times higher than those achieved following a one gram IV infusion of TXA (200 micrograms per milliliter [mcg/mL] vs 66 mcg/mL, respectively).^28^,^29^ Notably, the salival drug concentration remained at a therapeutic level, typically described to be in the range of 10–15 mcg/mL, for at least two hours following the rinse.^30^ In contrast, TXA levels in saliva following administration of an oral dose were found to be undetectable.^29^ The concept that topical application of TXA produces superior and sustained oral tissue drug concentration inclines us to reason that nebulized application of TXA has the potential to yield significant oropharyngeal drug concentrations as well.

Solomonov and colleagues reported the first case series of nebulized TXA, at a dose of 500 mg, in four patients with hemoptysis. All four patients experienced cessation of bleeding following initial dose administration.^20^ Additional case reports and case series followed, using TXA for management of hemoptysis at doses ranging from 500 mg to 1000 mg, all reporting successful control of bleeding.^21^–^23^ The duration of therapy generally varied from single-dose administration to scheduled dosing for two to seven days. Scheduled dosing administration intervals ranged from every six hours to every 12 hours. There was considerable variability in the concentration of TXA solution used, ranging from 10–100 mg/mL.

The first blinded, randomized controlled trial to formally evaluate the use of nebulized TXA for treatment of hemoptysis was published in 2018 by Wand and colleagues.^31^ Despite a relatively small sample size of 47 patients, the trial showed impressive results with control of bleeding achieved in 96% of patients in the TXA group, compared to 50% in the placebo arm. It allowed for administration of nebulized TXA 500 mg three times daily for up to five days with exact duration determined by the treating physician. The only adverse event attributed to the inhaled antifibrinolytic was a single report of bronchospasm, which was successfully managed with inhaled bronchodilators.

In the pediatric population, Bafaqih and colleagues conducted a pilot study exploring the feasibility and efficacy of using nebulized TXA for diffuse alveolar hemorrhage in mechanically ventilated pediatric patients.^32^ A dose of TXA 250 mg (for weight less than 25 kilograms) to 500 mg (for weight greater than 25 kilograms) was administered every six hours. Of the 18 patients enrolled in the study, 10 (55.6%) responded to nebulized TXA therapy within 24 hours. More recently, O’Neil and colleagues reported a seven-year retrospective observational review of nebulized or endotracheally instilled TXA for pulmonary hemorrhage in 19 pediatric intensive care unit patients.^33^ The use, dosing, and frequency of administration of the nebulized TXA was determined by the treatment team in each case and varied between 250–500 mg every six to 24 hours, with the most frequent dosing interval being every eight hours. A single administration is reported to have been made via direct endotracheal installation and all but one of the patients in the study were mechanically ventilated. Improvement in pulmonary hemorrhage was reported in nearly all cases following the initial dose of TXA, and 18 patients (95%) achieved cessation of bleeding within 48 hours (primary outcome). Neither study reported occurrence of side effects related to the inhaled TXA.

Successful use of aerosolized TXA has been reported in the setting of epistaxis as well. Booth and colleagues reported the use of nebulized TXA 500 mg in a patient with recent septoplasty who had presented with bilateral epistaxis and posterior oropharyngeal blood. The patient was instructed to breathe through her nose during the 15-minute TXA nebulization to maximize drug delivery into the nasal passages. Cessation of bleeding was observed within 15 minutes of administration of the full dose and was maintained until the patient’s discharge four hours later.^26^ Heymer and colleagues described an alternative method of local application of TXA for management of posterior nasal bleeding via mucosal atomizer device, in lieu of posterior nasal packing.^24^ They propose a dose of 200 mg (2 mL) of TXA applied into the affected nostril using a commercially available mucosal atomizer and suggest a repeat dose if bleeding persists after three minutes following the initial application.

In the setting of tonsillectomy, various studies have evaluated the use of TXA in the pre-, peri- and immediate post-operative settings with mixed results on operative blood loss and subsequent bleeding rates.^34^ Of those that have evaluated postoperative bleeding rates, a distinction between primary and secondary events has not always been made.^35^ Importantly, all the studies that have involved the use of TXA in the postoperative period have aimed to assess its potential prophylactic effect on preventing post-tonsillectomy hemorrhage, which may have different outcomes when compared to the drug’s application for management of active bleeding.

In 2012, Chan and colleagues performed the first systematic review and meta-analysis of the use of TXA in the setting of tonsillectomy, and their work has been cited as having concluded that there is no benefit of TXA on post-tonsillectomy hemorrhage.^35^,^36^ However, six of the seven studies included in the meta-analysis did not use the drug beyond the immediate postoperative period. Given TXA’s relatively short half-life of three hours and the reversible nature with which it inhibits fibrinolysis, it is unlikely that its effects would persist beyond a few to several hours after the last dose. Thus, it would be unsurprising that TXA administration prior to discharge offers no benefit on secondary bleeding rates. The remaining study did extend the use of TXA for four days postoperatively; however, it used the drug in its oral form. It has since been demonstrated that oral administration of TXA does not achieve detectable drug concentration in saliva and so would not be suitable for this application.

Hinder and Tschopp evaluated the use of TXA 0.2% solution applied topically, via gargle or spray at the tonsillar fossa, five to six times daily on postoperative days 5–10.^37^ They found no difference in secondary bleeding rates between the 246 patients in the TXA group and a historic control cohort (19% vs 22%, respectively). A tendency toward lower rates of bleeding requiring surgical intervention was noted in the treatment arm (8.9% vs 11.3%). This study provides the most convincing evidence for a lack of prophylactic benefit on secondary bleeding rates, although the finding may be somewhat unsurprising given that secondary tonsillectomy bleeding events typically result from mechanical displacement or sloughing of tonsillar eschar. The authors’ findings do, however, suggest that there may be a small benefit of prophylactic topical TXA on severity of bleeding and need for repeat surgical intervention in patients older than 12 years. The reproducibility of this finding and its clinical implications merit further evaluation via prospective randomized controlled trial.

No publication to date has directly studied the effect of TXA, in any formulation, on management of acute secondary post-tonsillectomy bleeding. No specific method for nebulization has been previously proposed in the context of TXA use for tonsillar bleeding. Without available evidence from controlled trials it is impossible to deduce the optimal dose, dilution, and administration method. However, there are some general considerations that could be applied to optimize drug delivery to the tonsillar region in this setting.

The amount of nebulized solution deposited within the respiratory system is influenced by the size of the aerosolized particles. Aerosol particles larger than 15 micrometers (μm) generally deposit in the mouth and nose. Particles in the range of 10–15 μm reach the upper airways, and those smaller than 10 μm and 5 μm reach the large bronchi and lower airways, respectively.^38^ Thus, to achieve higher drug concentration at the tonsillar region and minimize drug delivery to the lower airways, programming nebulizer settings to deliver aerosol particles in the range of 10–15 μm would be desired. This is, of course, in contrast to the approach applied to nebulized bronchodilator medications where the goal is to generate aerosol particles smaller than 5μm to reach the large bronchi and alveoli of the lower respiratory system. Specific settings vary between nebulizer brands and types, but larger aerosol particles are generally achieved using a lower gas-flow rate or lower pressure. Whenever possible, the use of a mouthpiece would be preferred over the use of a facemask as the former decreases the amount of aerosol deposited onto the nose, eyes, and face.^38^

The “dead space” of the nebulizer should also be taken into consideration, as liquid occupying it would not be nebulized. Available data suggest that increasing nebulizer fill volume decreases the amount of drug that remains trapped in the delivery system.^38^,^39^ Thus, we suggest that TXA doses be diluted to at least the recommended fill volume (typically 4–5 mL) of the nebulizer chamber. This may be particularly important with smaller doses, which may be used in younger patients.

## CONCLUSION

A hemostatic benefit in tonsillar bleeding following nebulized TXA administration was observed in 75% of the patients with active hemorrhage in this small case series. Given the limited therapeutic options for managing tonsillar bleeding in the ED, the use of nebulized TXA may be a reasonable, safe, non-invasive option for short-term stabilization pending definitive surgical intervention. Nebulization of the drug offers the advantage of simple and fast delivery of the antifibrinolytic to the targeted tissue site while requiring minimal patient cooperation. Future randomized controlled trials would be valuable to better assess the effectiveness of TXA in patients with post-tonsillectomy hemorrhage and provide insight into remaining questions including optimal dosing and dilution method, optimal duration of administration, the role of repeat or scheduled dosing, and the potential for synergy with other agents (eg, racemic epinephrine).

## Figures and Tables

**Table:** Characteristics, interventions, and outcomes of patients with post-tonsillectomy bleeding treated with nebulized tranexamic acid.

Case #	Age/gender	POD	TXA dose	Dilution	Doses given in ED	Hemostasis achieved in ED?	Additional interventions	Need for OR?
1	43/F	6	1000 mg	none	1	Yes	Direct pressure with TXA-soaked gauze, nebulized racemic epinephrine	Yes
2	13/F	7	500 mg	1:1 normal saline	1	No	Ice water gargle	Yes
3	28/M	9	500 mg	none	1	Yes	None	No*
4	17/M	5	500 mg	none	1	Temporarily	Ice water gargle	Yes
5	23/M	13	500 mg	none	1	No	Ice water gargle, clot suction, atomized TXA, topical oxymetazoline, topical lidocaine, lidocaine/epinephrine injection, direct pressure	Yes
6	6/M	6	500 mg	none	1	Yes	Ice water gargle	Yes
7	13/F	12	500 mg	1:1 normal saline	1	Yes	None	Yes
8	24/M	15	1000 mg	none	1	Yes	None	Yes

* Patient had previously required return to the OR on POD four due to postoperative bleeding.

*POD*, postoperative day; *TXA*, tranexamic acid; *F*, female; *M*, male; *mg*, milligram; *ED*, emergency department; *OR*, operating room.
